# Union, complication, reintervention and failure rates of surgical techniques for large diaphyseal defects: a systematic review and meta-analysis

**DOI:** 10.1038/s41598-022-12140-5

**Published:** 2022-06-01

**Authors:** Pietro Feltri, Luca Solaro, Alessandro Di Martino, Christian Candrian, Costantino Errani, Giuseppe Filardo

**Affiliations:** 1 Service of Orthopaedics and Traumatology, Department of Surgery, EOC, 6900 Lugano, Switzerland; 2 Clinica Ortopedica e Traumatologica II, IRCCS Istituto Ortopedico Rizzoli, Via Pupilli, 1, 40136 Bologna, Italy; 3 Faculty of Biomedical Sciences, Università Della Svizzera Italiana, Via Buffi 13, 6900 Lugano, Switzerland; 4 Orthopaedic Service, Musculoskeletal Oncology Department, IRCCS Istituto Ortopedico Rizzoli, 40136 Bologna, Italy; 5 Applied and Translational Research Center, IRCCS Istituto Ortopedico Rizzoli, 40136 Bologna, Italy

**Keywords:** Surgical oncology, Trauma

## Abstract

To understand the potential and limitations of the different available surgical techniques used to treat large, long-bone diaphyseal defects by focusing on union, complication, re-intervention, and failure rates, summarizing the pros and cons of each technique. A literature search was performed on PubMed, Web of Science, and Cochrane databases up to March 16th, 2022; Inclusion criteria were clinical studies written in English, of any level of evidence, with more than five patients, describing the treatment of diaphyseal bone defects. The primary outcome was the analysis of results in terms of primary union, complication, reintervention, and failure rate of the four major groups of techniques: bone allograft and autograft, bone transport, vascularized and non-vascularized fibular graft, and endoprosthesis. The statistical analysis was carried out according to Neyeloff et al., and the Mantel–Haenszel method was used to provide pooled rates across the studies. The influence of the various techniques on union rates, complication rates, and reintervention rates was assessed by a z test on the pooled rates with their corresponding 95% CIs. Assessment of risk of bias and quality of evidence was based on Downs and Black’s “Checklist for Measuring Quality” and Rob 2.0 tool. Certainty of yielded evidence was evaluated with the GRADE system. Seventy-four articles were included on 1781 patients treated for the reconstruction of diaphyseal bone defects, 1496 cases in the inferior limb, and 285 in the upper limb, with trauma being the main cause of bone defect. The meta-analysis identified different outcomes in terms of results and risks. Primary union, complications, and reinterventions were 75%, 26% and 23% for bone allografts and autografts, 91%, 62% and 19% for the bone transport group, and 78%, 38% and 23% for fibular grafts; mean time to union was between 7.8 and 8.9 months in all these groups. Results varied according to the different aetiologies, endoprosthesis was the best solution for tumour, although with a 22% failure rate, while trauma presented a more composite outcome, with fibular grafts providing a faster time to union (6.9 months), while cancellous and cortical-cancellous grafts caused less complications, reinterventions, and failures. The literature about this topic has overall limited quality. However, important conclusions can be made: Many options are available to treat critical-size defects of the diaphysis, but no one appears to be an optimal solution in terms of a safe, satisfactory, and long-lasting outcome. Regardless of the bone defect cause, bone transport techniques showed a better primary union rate, but bone allograft and autograft had fewer complication, reintervention, and failure rates than the other techniques. The specific lesion aetiology represents a critical aspect influencing potential and limitations and therefore the choice of the most suitable technique to address the challenging large diaphyseal defects.

## Introduction

Large diaphyseal defects (LDD) of long bones are a complex and relatively common clinical problem in orthopaedic surgery. LDD are by definition considered incapable of spontaneous healing and therefore represent an indication for surgery, accounting for millions of surgical procedures per year^1^. Bone loss can be the result of a variety of aetiologies: high-energy trauma, tumour resection, congenital defects, bone resection for non-union, necrosis, and osteomyelitis^2^. Reconstruction of LDD remains a surgical challenge due to healing difficulties, associated lesions, and the high risk of complications and need for reinterventions. The issues with reconstructing segmental defect are related to the structural and functional importance of long bones, and the high mechanical stress forces involved, particularly for the inferior limb when subjected to weight-bearing. Moreover, LDD can be accompanied by concomitant soft tissue damage and infection^2^.

LDD have been increasingly studied and several options have been proposed to address this challenge. Bone autografts and allografts were, historically, the first treatments to be developed^3–6^. Vascularized fibular grafts were then introduced to increase osseointegration and vitality of the reconstructed bone^7^. In the last decades, bone transport and distraction osteogenesis increased their popularity and, more recently, novel internal lengthening techniques, combined autograft-allograft reconstructions, titanium mesh cages and bioactive membranes were introduced to allow complex biological reconstructions^8–10^. Intercalary endoprosthesis reconstructions are also possible, particularly when rapid recovery is preferred over long-term durability^11^. Despite the efforts and advancements in surgical techniques, a consensus on the best surgical approach for long bone diaphysis defects has not been reached, yet. LLD reconstruction impacts heavily on patients, with often long and painful recovery and uncertain outcomes. Thus, it is of outmost importance to understand pros and cons of each option and properly chose the best treatment strategy for each patient.

The aim of this systematic review and meta-analysis was to understand potential and limitations of the different available surgical techniques used to treat large long-bone diaphyseal defects by focusing on union, complication, re-intervention, and failure rates.

## Materials and methods

### Literature research

A review protocol was created according to the preferred Reporting Items for Systematic Reviews and Meta-Analyses (PRISMA) statement (www.prisma-statement.org). A comprehensive search was performed in the bibliographic databases PubMed, Web of Science, Embase, and Wiley Cochrane Library from inception up to 16 March 2022. The following terms were used “(diaphyseal OR segmental OR intercalary) AND bone defect AND treatment”, no filters of any type were applied, and the choice “all fields” was applied, when relevant Inclusion criteria were: patients, both male and female, with a diagnosis of segmental bone defects in the diaphysis of the long bones, undergoing surgical treatment of any type for these defects. Comparative and non-comparative studies, with no limitations on the follow-up were included. Case reports or case series describing ≤ 5 cases and articles in languages other than English were excluded. Preclinical and ex vivo studies, studies involving mixed series with not only diaphysis defects, and review articles were also excluded. The purpose of the study was to analyse the main outcomes (primary union, complications, re-interventions, and failures of the different available surgical techniques used to treat large long-bone diaphyseal defects.

### Data extraction

**Figure 1:**
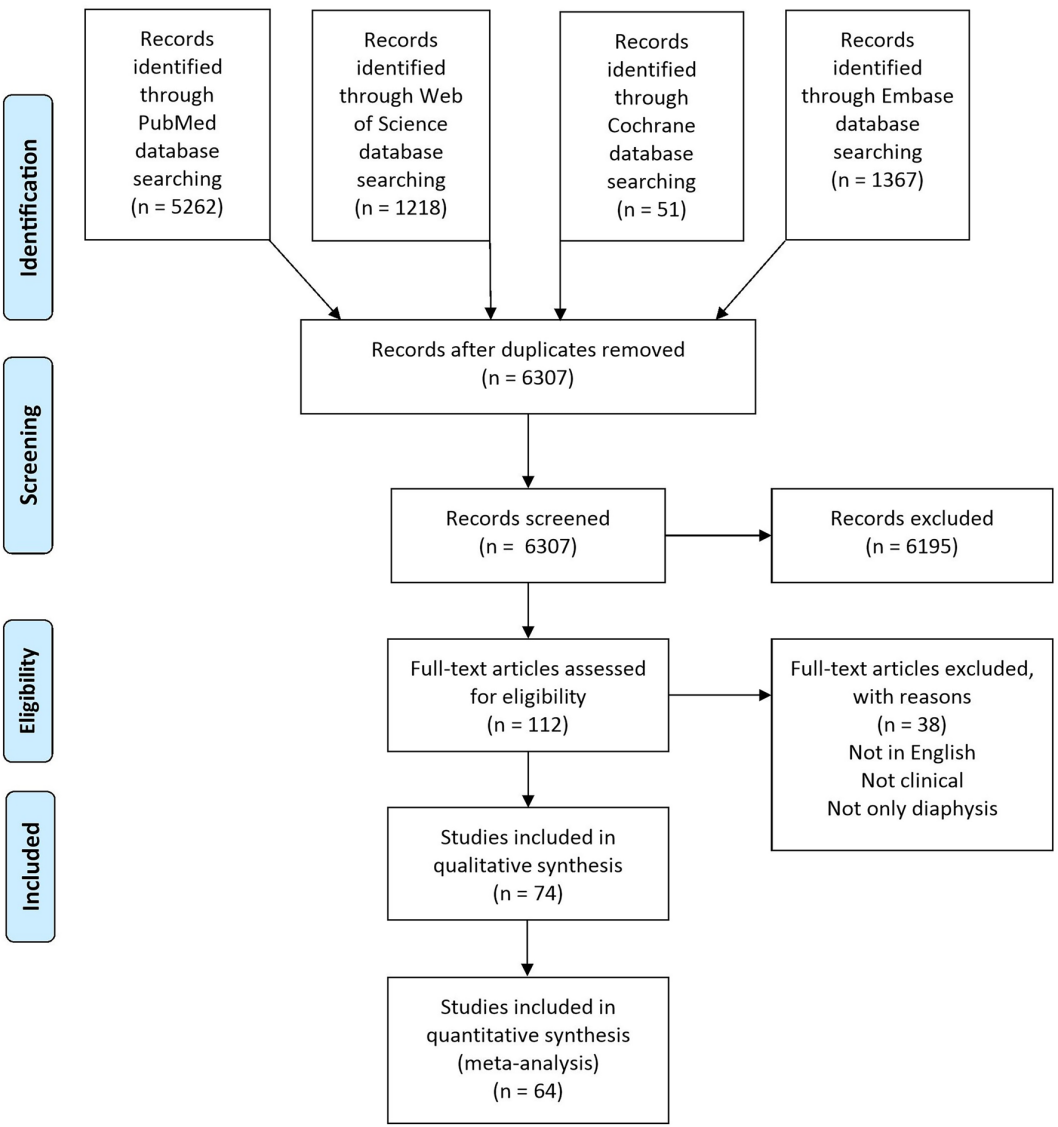
PRISMA (Preferred Reporting Items for Systematic Meta-Analyses) flowchart of the study selection process.

Two independent reviewers (PF, LS) screened all articles on the title and abstract and whether they met the inclusion criteria. After the first screening, the articles that met the inclusion criteria were evaluated on full-text eligibility and were excluded if they met one of the exclusion criteria (Fig. 1). In case of disagreement between the two reviewers (PF, LS) a third reviewer was consulted to reach a consensus (CC).

Data were independently extracted on a preconceived data extraction form using Excel (Microsoft). The following data were extracted: first author, journal, year of publication, level of evidence, population characteristics, cause of bone loss, surgical technique, graft characteristics, fixation method, surgery time, union rate, complications, reintervention, time to full weight-bearing, functional outcomes, and amputation. In case of missing data, an attempt to contact the corresponding author was made; in case of studies with data upon request, they were asked to the corresponding author. Delayed union was defined as the failure to reach bone union within 6 months after reconstruction, whereas non-union was defined as the failure to reach bone union at the time of the last follow-up after 6 months^12^. After independent data collection, the reviewers compared the extracted data.

Included articles were sorted in homogenous groups based on the surgical technique used. The interventions were classified into four main groups according to the treatment strategy: vascularized and non-vascularized fibular graft, bone autograft and allograft, bone transport, endoprosthesis. Comparable outcomes were also analysed between different groups of techniques, and their results were descriptively discussed. A meta-analysis was performed focusing on union, complication, re-intervention, and failure rates.

### Assessment of risk of bias and quality of evidence

The Downs and Black’s “Checklist for Measuring Quality”^13^ and the Cochrane Collaboration Risk of Bias (RoB) 2.0 tool was used to evaluate the risk of bias^14^. The first contains 27 ‘yes’-or-'no’ questions across five sections; and provides a numeric score out of a scale of 32 points. The five sections include questions about the overall quality of the study (10 items), the ability to generalize the findings of the study (3 items), the study bias (7 items), the confounding and selection bias (6 items), and the power of the study (1 item). Rob 2.0 is designed into a fixed set of bias domains, focusing on different aspects of trial design, conduct, and reporting. Within each domain, a series of questions ask information about features of the trial relevant to the risk of bias. A proposed judgement about the risk of bias arising from each domain is generated by an algorithm, based on answers to these questions. The risk of bias can be judged as 'Low', 'Some concerns', or 'High'. The quality of evidence for all outcomes was graded using the Grading of Recommendations Assessment, Development, and Evaluation, which classifies the quality of evidence as high, moderate, low, or very low^15^. Evidence from RCT will start at high quality and be selected to be downgraded by 1 or 2 levels depending on risk factors such as the risk of bias, imprecision, inconsistency, indirectness, and publication bias. Assessment of risk of bias and quality of evidence were completed independently for all outcomes by 2 authors (PF, LS) and a third author (CC) solved any possible discrepancy reaching consensus.

### Statistical analysis

The statistical analysis was carried out according to Neyeloff et al.^16^ using Microsoft Excel. The Mantel–Haenszel method was used to provide pooled rates across the studies. A statistical test for heterogeneity was first conducted with the Cochran Q statistic and I^2^ metric and was considered the presence of significant heterogeneity with I2 values ≥ 25%. When no heterogeneity was found with I2 < 25%, a fixed-effect model was used to estimate the pooled rates and 95% CIs. Otherwise, a random-effect model was applied, and an I^2^ metric was evaluated for the random effect to check the correction of heterogeneity. The studies' rate confidence intervals were carried out using the continuity-corrected Wilson interval. The influence of the various techniques, as divided into the four groups, on union rates, complication rates, and reintervention rates was assessed by a z test on the pooled rates with their corresponding 95% CIs. Descriptive statistics were performed to describe the sociodemographic and injury-related characteristics of the patients included in the retrieved studies. Continuous variables were expressed as pooled means with their confidence intervals and standard deviation, using Excel (Microsoft).

## Results

### Study selection

**Figure 2:**
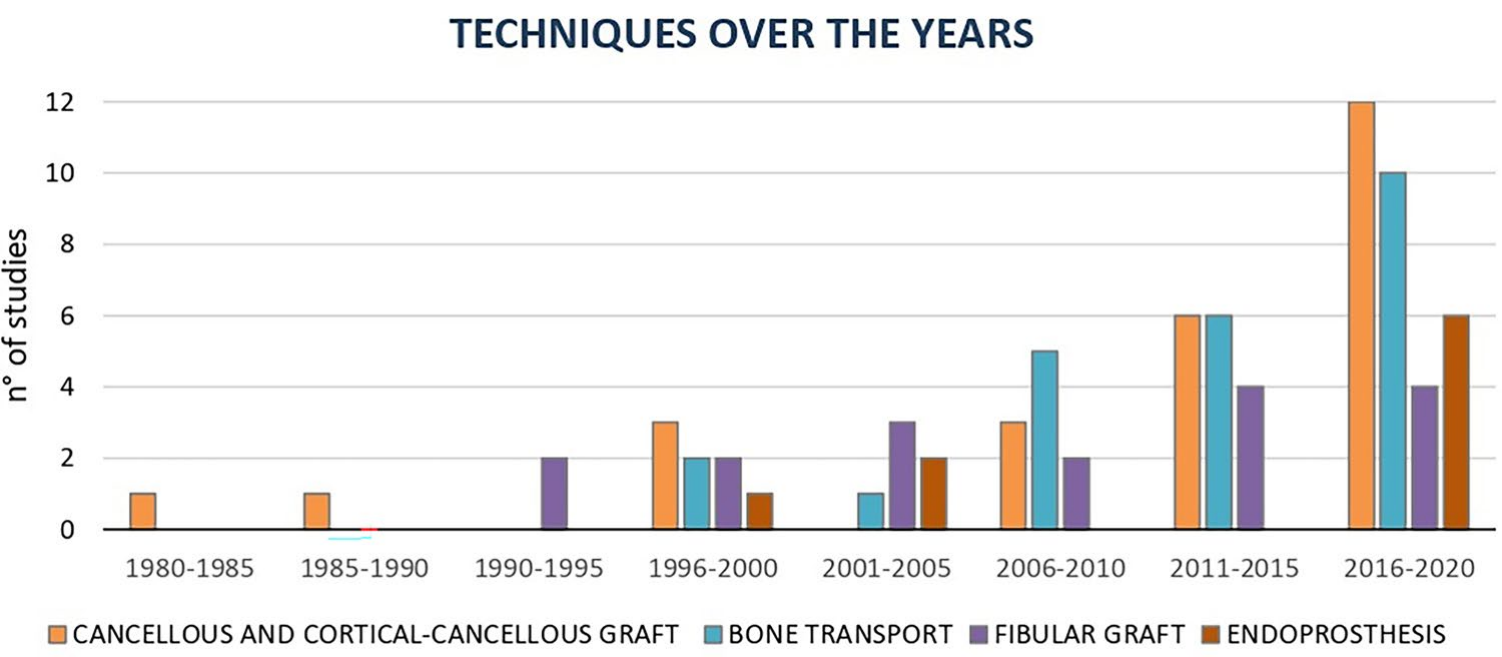
Graphic representation of the trend, over the years, of the four major groups of techniques.

A total of 7903 articles were retrieved after a search on PubMed, Web of Science, Embase, and Cochrane Library databases. After removal of duplicates, screening of the title and abstract, and full-text assessment, 74 articles were included for the quantitative synthesis. A summary of the study selection process is shown in the PRISMA flow diagram (Fig. 1). The studies were published between 1983 and 2022, thus showing results obtained in the last 39 years, with an increasing number of studies on the treatment of diaphyseal bone defects over time (Fig. 2). Regarding the level of evidence, one study was a Level two randomized controlled trial, seven were Level three comparative studies, and 66 were Level four case series. A summary of all study characteristics is shown in Table 1. Four main groups of reconstruction techniques were evidenced: Bone allograft and autograft (27 study arms), bone transport techniques (25 study arms), vascularized and non-vascularized fibular graft (19 study arms), and endoprosthesis (9 studies). Among them, six studies presented different techniques and were analysed for each technique within the corresponding category. Three studies presented data that were not possible to categorize homogenously, and seven studies presented data that could not be used for the meta-analysis. Thus, a meta-analysis was performed on 64 of the included studies for the union, complication, reintervention, and failure rate. Other aspects could not be used for the meta-analysis because of heterogeneity or lack of data.

**Table 1:** Summary of all the studies characteristics. VFG (vascularised fibular graft), NVFG (non-vascularised fibular graft), BTON (Bone Transport Over Intramedullary Nail), PMMA (Poly Methyl Methacrylate), IMT (Induced Membrane Technique).

Article	Study design	F-UP	N° of PTS (M:F)	Aetiology	Technique	Defect (CM)
Haque^41^	Case series	NA	20 (16:4)	Trauma	Hemicylindrical sliding bone graft, bone graft, tibio-fibular synostosis	3.5
Christian^17^	Case series	27	8 (8:0)	Trauma	Massive autogenous cancellous bone graft	10.2
Banic^66^	Case series	17.3	7 (NA)	Trauma	VFG: double strut VFG + cancellous bone graft	11.6
Herte^67^	Case series	32	12 (9:3)	Trauma, Tumor	VFG + cancellous bone graft	12
Abudu^82^	Case series	65	18 (14:4)	Tumor	Endoprosthesis	21
Khan^68^	Case series	90	8 (5:3)	Trauma	VFG + cancellous bone graft	5
Hsu^69^	Case series	36	30 (16:4)	Tumor	VFG + corticocancellous bone graft	12.9
Barbieri^18^	Case series	NA	12 (10:2)	Trauma	Corticocancellous bone autograft	NA
Polyzois^61^	Case series	38	42 (29:13)	Trauma, Osteomyelitis, Congenital, Nonunion	Ilizarov distraction osteogenesis	6
Smrke^63^	Case series	115	20 (17:3)	Infection	Ilizarov distraction osteogenesis, Ljubljana traction	10.4
Ueng^42^	Case series	58	15 (14:1)	Nonunion	Corticocancellous bone autograft, VFG	NA
Ring^19^	Case series	31	15 (9:6)	Nonunion	Autogenous bone graft	3
Morsi^70^	Case series	32.5	7 (6:1)	Nonunion	NVFG	4.7
Taha^80^	Case series	59.9	8 (6:2)	Tumor, Trauma, Osteomyelitis	NVFG	10.4
Aldlyami^84^	Case series	107	35 (22:13)	Tumor	Endoprosthesis	19
Chang^71^	Case series	24	14 (NA)	Tumor	VFG + intercalary allograft	NA
El-Mowafi^52^	Case series	27	16 (12:4)	Infection	Ilizarov distraction osteogenesis	6.4
Ahlmann^83^	Case series	21.6	6 (4:2)	Tumor	Endoprosthesis	12.3
Jones^20^	RCT	12	30 (NA)	Trauma	Allograft + BMP-2, autograft	3.8
Kocaoglu^54^	Case series	47.3	13 (8:5)	Osteomyelitis	Bone transport over intramedullary nail (BTON)	10
Catagni^48^	Case series	70.8	7 (6:1)	Osteomyelitis, Trauma, Infection	Ilizarov distraction osteogenesis	15.1
Moran^72^	Case series	36	7 (5:2)	Tumor	Capanna technique	13
Oh^57^	Case series	NA	12 (12:0)	Osteomyelitis	Bone transport over intramedullary nail (BTON)	5.8
Chaddha^49^	Case series	23.5	25 (25:0)	Trauma	Ilizarov distraction osteogenesis	8.9
Gupta^21^	Prospective	NA	23 (15:8)	Nonunion	Modified Nicoll's technique	4.7
Hutson^63^	Case series	58	18 (13:5)	Trauma	Bone transport	9.4
Prasarn^22^	Case series	60	15 (9:6)	Nonunion	Tricortical iliac crest bone graft	2.1
Li^73^	Case series	34.1	11 (5:6)	Tumor	Capanna technique	12.1
Liodakis^55^	Case series	61.2	22 (17:5)	Trauma	Monorail technique	7.4
Ruggieri^33^	Case series	29	24 (11:13)	Tumor	Modular intramedullary segmental defect fixation system	10
Li^74^	Case series	27.7	7 (4:3)	Tumor	Capanna technique	10.6
Karger^23^	Case series	NA	84 (79:5)	Trauma	Induced membrane technique	6.8
De Gauzy (2012)	Case series	NA	27 (17:10)	Trauma	Induced membrane technique, bone transport, autograft, VFG	NA
Puri^34^	Case series	34	32 (24:8)	Tumor	Resection, irradiation and reimplantation of bone	19
Toros^81^	Case series	37	6 (3:3)	Nonunion	VFG	NA
Farfalli^24^	Case series	73	26 (13:13)	Tumor	Intercalary allograft	NA
Borzunov^47^	Case–control	NA	83 (54:29)	Trauma, Osteomyelitis, Congenital, Tumor	Ilizarov distraction osteogenesis, gradual tibialisation of the fibula	12.8
Prejbeanu^32^	Case–control	30	12 (6:6)	Tumor	Intramedullary nail + PMMA	9
Xu (2013)	Case series	29	30 (21:9)	Nonunion	Ilizarov distraction osteogenesis	6.4
El Ghaffar^25^	Prospective	24	12 (12:0)	Trauma	Two-stage reconstruction: debridement + pin, then corticocancellous bone grafting	NA
Saka^26^	Case series	32	8 (5:3)	Nonunion	(Modified Nicoll's technique)	1.8
Schuh^75^	Case series	52	53 (26:27)	Tumor	VFG, NVFG	NA
Le Thua^76^	Case series	NA	26 (18:8)	Trauma, Osteomyelitis, Nonunion	VFG	10.8
Ajmera^44^	Case series	15	25 (25:0)	Trauma	Bone transport	5.5
Bernstein^46^	Case series	33	58 (39:19)	Trauma	Ilizarov distraction osteogenesis, bone transport over intramedullary nail (BTON)	5.3
Marais^58^	Case series	28	7 (NA)	NA	Bone transport	7
Davis^27^	Case series	42	7 (5:2)	Nonunion	Staged reconstruction technique (allograft + IM nail)	4.9
Gupta^28^	Case series	21.5	9 (7:2)	Nonunion	Induced membrane technique	5.2
Benevenia^88^	Case series	14	41 (27:14)	Tumor	Endoprosthesis	NA
Sadek^35^	Case–control	NA	30 (24:6)	Nonunion	Two-steps debridment + iliac graft vs one-step Ilizarov distraction osteogenesis	4.6
Huang^85^	Case series	9	16 (6:10)	Tumor	Intercalary endoprosthesis	10.2
Tong^36^	Case–control	25.3	39 (30:9)	Osteomyelitis	Induced membrane technique, Ilizarov distraction osteogenesis	6.8
Zoller^29^	Case series	18.3	9 (8:1)	Trauma	Induced membrane technique	6.4
Ferchaud^53^	Case series	62	7 (NA)	Trauma	Bone transport over intramedullary nail (BTON)	7.2
Tedesco^86^	Case series	39	6 (3:3)	Tumor	Endoprosthesis	NA
Attias^30^	Case series	55	17 (14:3)	Trauma	Cancellous bone graft (Titanium mesh cage)	8.4
Cano-Luis^77^	Case series	166.8	14 (13:1)	Trauma	VFG	NA
Zaho^90^	Case series	8.6	9 (4:5)	Tumor	Endoprosthesis	7.8
Errani^78^	Case series	96	81 (56:25)	Tumor	Massive bone allograft + VFG	15.9
Davda^51^	Case series	NA	10 (8:2)	Nonunion	Bone transport over intramedullary nail (BTON)	7
Meselhy^59^	Prospective	40.5	14 (10:4)	Osteomyelitis, Trauma	Ilizarov distraction osteogenesis	13.2
Catagni^50^	Case–control	43.3	86 (77:9)	Trauma, Nonunion, Osteomyelitis	Bifocal fibular transfer, trifocal fibular transfer	13
Liu^79^	Case series	65.1	15 (9:6)	Tumor	VFG + autograft	19.8
Salunke^37^	Case series	30.9	28 (NA)	Tumor	NVFG, extracorporeal radiotherapy autograft	14.9
Zheng^87^	Case series	13.7	49 (23:26)	Tumor	Endoprosthesis	9.2
Ma^31^	Case series	19.1	51 (32:19)	Trauma	Cancellous wrap + titanium mesh cage/line mesh/line-binding, IMT	5.9
Bas^45^	Case series	25.7	40 (26:14)	Trauma, Nonunion	Bone transport over intramedullary nail (BTON)	7.1
Lu^56^	Case series	25.8	12 (10:2)	Trauma, Nonunion	Bone transport	6.7
Choi^38^	Case series	18	8 (4:4)	Trauma	Autologous iliac graft	2.6
Lotzien^39^	Case series	33.1	31 (30:1)	Nonunion	Induced membrane technique	8.3
Huang (2021)	Comparative	29.1	77 (54:23)	Trauma, Osteomyelitis	Bone transport + graft + internal fixation / bone transport	13.5
Wang^40^	Case series	NA	42 (17:25)	Trauma	Induced membrane technique	6.3
Büyükdoğan^89^	Case series	17	22 (15:7)	Tumor	Endoprosthesis	10
Liu^65^	Case series	28.2	12 (10:2)	Osteomyelitis	Bone transport	5.1

### Patients and treatments characteristics

A total of 1781 patients were included in the analysis: 1496 cases with inferior limb reconstruction and 285 cases with upper limb reconstruction (tibia 60.0%, femur 24.5%, humerus 7.1%, radius 4.7%, and ulna 3.7%). While 15 articles reported mixed series of both inferior and upper limbs, 13 articles focused only on the upper limb reconstruction and 46 articles presented only results on the inferior limb. The average bone defect was 9.0 cm (range 1.6–31 cm). The aetiology of the defects included trauma in 751 cases (42.2%), tumour in 554 cases (31.1%), non-union after previous treatment in 289 cases (16.2%), infection in 177 cases (9.9%), and congenital defect in ten cases (0.6%). Aetiology of the defects are shown in Table 2. Gender was represented by 70.4% men and 29.6% women, and age presented a range of 2–86 years. The mean follow-up was 40.9 months (range 1 to 157 months). Results obtained for each treatment group are analysed in detail in the following paragraphs. Table 3 shows a summary of the results. Moreover, data are also reported quantifying the primary union, time to union, complication, reintervention, and failure rates of the different treatments based on the main aetiology subgroups trauma and tumour (see Tables 4 and 5 for details). A summary of complications is reported in Table 6.

**Table 2:** Summary of bone defect causes.

Treatment groups					
Causes of bone defect	Fibular graft (%)	Bone graft (%)	Bone transport (%)	Endoprosthesis (%)	Various techniques (%)
Tumor	73.1	18.8	1.0	100	0
Trauma	17.1	53.7	55.6	0	75.8
Nonunion	6.7	23.9	20.1	0	24.2
Infection	3.1	3.6	21.9	0	0
Congenital defect	0	0	1.4	0	0

**Table 3:** Summary of all outcomes.

Outcomes					
Groups of treatment	Primary union	Time to union	Complication	Reintervention	Failure
Bone allograft and autograft *27 studies, 564 patients*	75% (C.I. 72%–78%)	7.8 months (1–27 months)	26% (C.I. 22%–30%)	23% (C.I. 19%–28%)	8% (C.I. 6%–11%)
Bone transport *25 studies, 676 patients*	91% (C.I. 89%–93%)	8.9 months (3–52 months)	62% (C.I. 59%–65%)	19% (C.I. 16%–22%)	8% (C.I. 6%–10%)
Vascular and non-vascular fibular graft *19 studies, 327 patients*	78% (C.I. 73%–82%)	8.3 months (2–33 months)	38% (C.I. 33%–43%)	23% (C.I. 19%–28%)	8% (C.I. 5%–12%)
Endoprosthesis *9 studies, 202 patients*	NA	NA	26% (C.I. 21%–32%)	20% (C.I. 15%–28%)	22% (C.I. 18%–28%)

**Table 4:** Summary of trauma studies outcomes.

Trauma outcomes					
Groups of treatment	Primary union	Time to union	Complication	Reintervention	Failure
Bone allograft and autograft *12 studies, 294 patients*	89% (C.I. 85%–91%)	8.9 months (1–27 months)	22% (C.I. 18%–28%)	20% (C.I. 15%–27%)	1% (C.I. 1%–3%)
Bone transport *8 studies, 176 patients*	90% (C.I. 85%–93%)	9.8 months (4–22 months)	69% (C.I. 64%–74%)	42% (C.I. 34%–51%)	NA
Vascular and non-vascular Fibular graft *4 studies, 30 patients*	89% (C.I. 76%–96%)	6.9 months (2–33 months)	40% (C.I. 27%–56%)	41% (C.I. 27%-56%)	9% (C.I. 3%–24%)

**Table 5:** Summary of tumor studies outcomes.

Tumor outcomes					
Groups of treatment	Primary union	Time to union	Complication	Reintervention	Failure
Bone allograft and autograft *5 studies, 106 patients*	82% (C.I. 73%–88%)	NA	39% (C.I. 31%–48%)	31% (C.I. 23%–40%)	NA
Vascular and non-vascular fibular graft *9 studies, 246 patients*	74% (C.I. 68%–79%)	8.4 months (2–27 months)	38% (C.I. 33%–44%)	26% (C.I. 21%–31%)	13% (C.I. 9%–19%)
endoprosthesis *9 studies, 202 patients*	NA	NA	26% (C.I. 21%–32%)	20% (C.I. 15%–28%)	22% (C.I. 18%–28%)

**Table 6:** Summary of all complications.

Groups of treatment					
Complications	Fibular graft	Bone graft	Bone transport	Endoprosthesis	Various techniques
Fracture	23.9% (31)	4.9% (9)	3.8% (16)	11.3% (8)	2.7% (1)
Infection	11.5% (15)	28.0% (51)	24.7% (105)	7.1% (5)	10.8% (4)
Donor site morbidity	2.3% (3)	2.7% (5)	1.4% (6)	0% (0)	0% (0)
Nonunion	16.7% (22)	25.3% (46)	9.6% (41)	0% (0)	21.6% (8)
Limb deformity	6.2% (8)	3.3% (6)	7.5% (32)	2.9% (2)	13.5% (5)
Vascular injury	0.8% (1)	1.6% (3)	0.7% (3)	0% (0)	0% (0)
Flap necrosis	2.3% (3)	4.4% (8)	0% (0)	0% (0)	8.2% (3)
Mechanical problems	4.6% (6)	8.2% (15)	4.7% (20)	64.3% (36)	0% (0)
Wound deiscency	11.5% (15)	3.3% (6)	6.6% (28)	2.9% (2)	0% (0)
Nerve palsy	2.3% (3)	0.5% (1)	4.0% (17)	4.3% (2)	13.5% (5)
Hematoma	0% (0)	1.1% (2)	0.9% (4)	0% (0)	0% (0)
Osteomyelitis	0.8% (1)	0% (0)	1.6% (7)	0% (0)	0% (0)
Pseudoarthrosis	0.8% (1)	0% (0)	2.6% (11)	0% (0)	0% (0)
Rom limitation	2.3% (3)	3.3% (6)	15.3% (65)	0% (0)	29.7% (11)
Chronic pain	3.9% (5)	0% (0)	2.4% (10)	0% (0)	0% (0)
Implant allergy	0% (0)	0% (0)	0.5% (2)	0% (0)	0% (0)
Limb length Discrepancy	4.6% (6)	3.8% (7)	8.2% (35)	2.9% (2)	0% (0)
Delayed union	5.4%(7)	9.3% (17)	6.1% (26)	4.3% (3)	0% (0)

### Bone allograft and autograft

Bone allograft and autograft was used in 27 study arms^17–43^ for a total of 564 patients (mean age 35.0 years): the inferior limb was involved in 78.2% of the cases, while the upper limb accounted for 21.8% of the cases. The defect was exclusively due to trauma in twelve studies, non-union in nine studies, tumour resection in five studies, and infection in one study. The average bone defect was 7.3 cm (range 0.5–27.8 cm), and the average follow-up was 33.3 months (range 6–180 months). A two-stage induced membrane technique (IMT) was used in seven studies, with massive autologous cancellous bone graft as a second step after the polymethacrylate spacer, while in nine studies cortical-cancellous iliac bone autograft was used, fixed either with external or internal fixation devices. In two studies autogenous cancellous bone graft was used alone, fixed with a plate and screws, while intercalary allograft with cancellous bone graft was used in two studies. Two studies reported the usage of titanium mesh cages with autologous bone graft, two studies the reimplantation of extracorporeal devitalized cancerous bone, one study reported the use of BMP-2, one study reported the application of multiple wrapped cancellous bone autograft methods, and another study reported a solution with an intramedullary nail and polymethacrylate spacer as a definitive treatment. Four studies did not report data on primary unions; in the others, the pooled union rate was 75% (C.I. 72%–78%). The average time to union was 7.8 months (range 1–27 months). Complications were reported 182 times, with a mean of 26% (C.I. 22%–30%) (details in Table 6). The reintervention rate was 23% on average (C.I. 19%–28%), and the failure rate was 8% (C.I. 6%–11%). Outcomes of trauma and tumor treatment are detailed in Tables 4 and 5, respectively. A specific analysis of the two-step Masquelet technique^28,29,36,39,40^ underlined a primary union rate of 53%, a complication rate of 15.0%, a reintervention rate of 27.2%, and a failure rate of 15.0%. Finally, in the studies on bone allografts or autografts used alone^17–20,22,24,25,30,33,42^ a pooled primary union rate of 87.0%, a time to union of 5.3 months, a complication rate of 31.0%, a reintervention rate of 26%, and a failure rate of 3.9% were reported.

### Bone transport

Bone transport with distraction osteogenesis has become over the years one of the most used techniques for bone regeneration. A total of 25 studies used a bone transport technique^35,36,43–65^ for a total of 676 patients (mean age 35.4 years). The inferior limb was involved in 98.1% of the cases, while the upper limb accounted for 1.9% of the cases. The defect was exclusively due to trauma in seven studies, infection in seven studies, to non-union in three studies, while eight studies reported mixed aetiology. The average bone defect was 8.8 cm (range 2.7–28 cm), and the average follow-up was 35.7 months (range 6–168 months). The Ilizarov method with external fixation was used in 15 studies^35,36,46–50,52,56,59–62,65^, intramedullary nailing and/or external fixation was used in eight studies^43,45,51,53–55,57,64^, a monolateral external fixation was used in one study^44^, and bone transport with a five-ring circular external fixator in one study^58^. One of the studies in the Ilizarov group was a case–control comparing bifocal versus trifocal fibular transfer, showing better primary union rate, fewer complications and reinterventions in the trifocal group. The primary union was obtained in 91% of the patients (C.I. 89%–93%); the mean external fixator time was 8.9 months (range 3–52 months). Complications were reported 425 times, with a pooled ratio of 62% (C.I. 59%–65%) but most of them were minor complications and did not require any invasive intervention (details in Table 6). The reintervention rate was 19% (C.I. 16%–22%), and the failure rate was 8% (C.I. 6%–10%). Outcomes of trauma treatment are detailed in Table 4.

A subgroup of six studies^45,46,50,51,53,54,57^ focusing on the bone transport approach showed a primary union rate of 89.7% with a mean external fixator time of 6.6 months (range 4.2–9.3 months), a complication rate of 66.9%, a reintervention rate of 29.8% and a failure rate of 3.7%.

### Vascular and non-vascular fibular graft

The fibular graft was the elective technique in 19 studies^37,42,43,66–81^, for a total of 327 patients (mean age 22.2 years): the inferior limb was involved in 81.3% of the cases, while the upper limb accounted for 18.7% of the cases. The defect was exclusively due to tumor resection in nine studies, trauma in four studies, and non-union after previous treatment in three studies, while three studies reported mixed aetiology. The average defect was 13.4 cm (range 1–25 cm). The average follow-up was 62.8 months (range 6–276 months). In all except four studies, the graft was a vascularized fibular graft, in some cases used in combination with cortical-cancellous bone autograft or allograft. The primary union was achieved in 78% of the patients (C.I. 73%–82%), in an average time of 8.3 months (range 2–33 months). Complications were reported 130 times, with a complication rate of 38% (C.I. 33%–43%) (details in Table 6). The reintervention rate was 23% on average (C.I. 19%–28%), and the failure rate was 8% (C.I. 5%–12%). Outcomes of trauma and tumor treatment are detailed in Tables 4 and 5, respectively. A subgroup of studies focused specifically on the Capanna technique^72,74^, reporting a primary union rate of 82.7%, in an average time of 7.6 months, a complication rate of 43.8%, a reintervention rate of 25.3%, and a failure rate of 8%. For non-vascular fibular grafts^37,70,75,80^ the mean primary union rate was 73.0%, with a complication rate of 49.0% and a reintervention rate of 42.0%.

### Endoprosthesis

Intercalary endoprosthesis was chosen as a solution to the diaphyseal bone defect in nine studies^82–90^, for a total of 202 patients (mean age 52.7 years). Bone defects involved the inferior limb in 54.5% and the upper limb in 45.5% of the time, and the aetiology of the defect was tumour resection in all the retrieved studies. The bone defect measured on average 13 cm (range 6–28 cm), and the follow-up of the studies was 35.2 months (range 1–306 months). A meta-analysis on the primary union rate was not performed due to the lack of data in the retrieved studies; however, it was possible to calculate the complication rate (mean 26%, C.I. 21%–32%), total complications 60, and the reintervention rate (mean 20%, C.I. 15%–28%). The meta-analysis on failure rate showed a mean of 22% (C.I. 18%–28%).

### Risk of bias and quality of evidence

**Figure 3:**
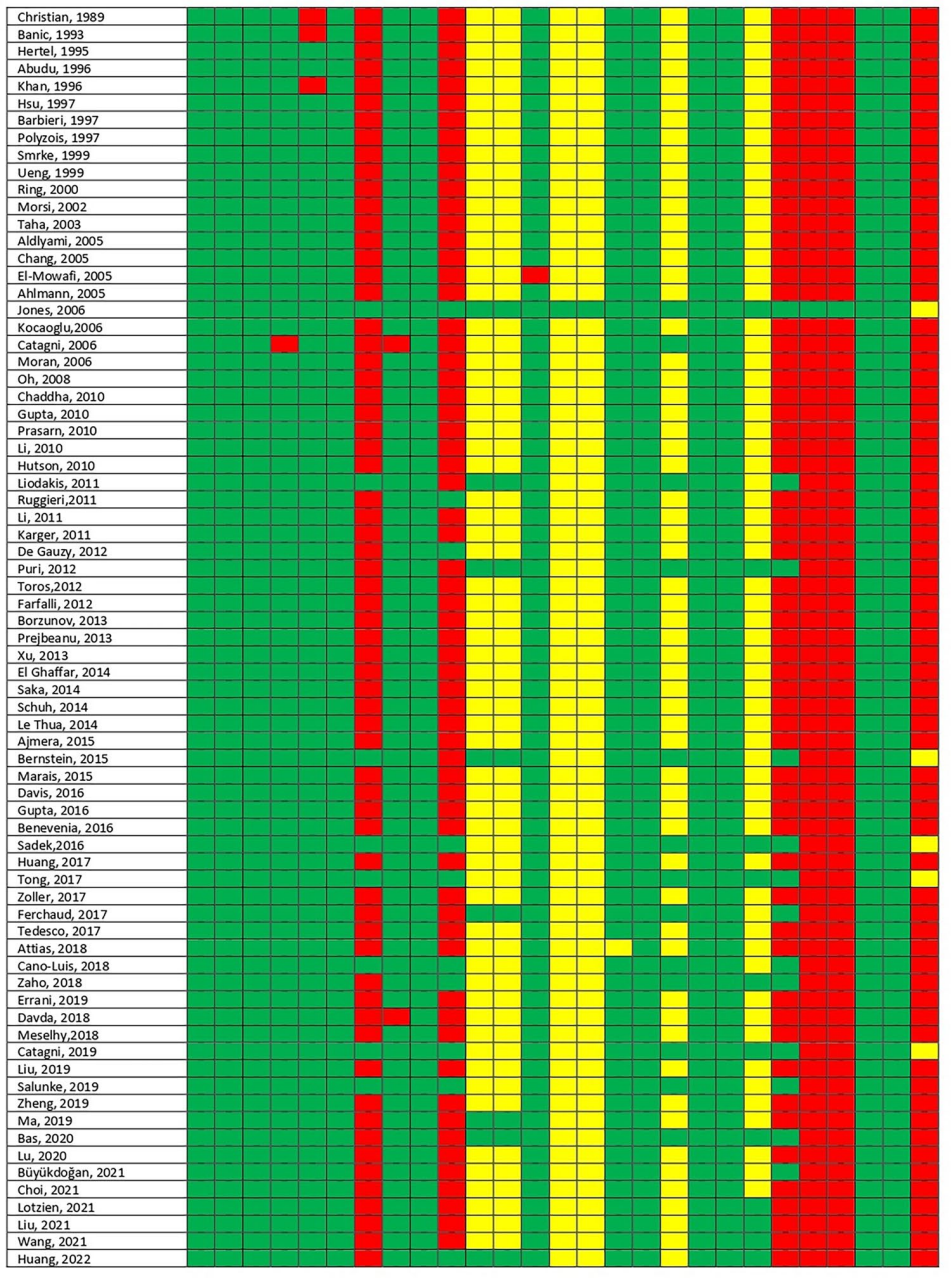
Risk of bias of all the included studies, evaluated in accordance with the “Downs and Black’s tool for assessing the risk of bias”.

**Figure 4:**
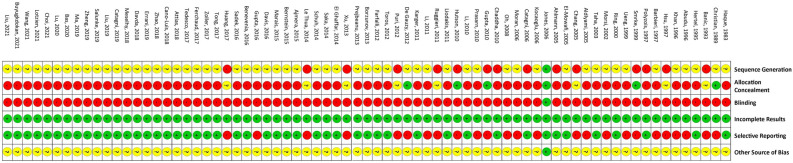
Risk of bias of all the included studies, evaluated in accordance with the Rob 2.0 tool.

The Downs and Black’s Checklist^13^ gives each study an excellent ranking for points ≥ 26, a good ranking for points between 20 and 25, a fair ranking for points between 15 and 19, and a poor ranking for a score ≤ 14 points. Accordingly, among the retrieved studies four studies were classified as poor, 50 studies as fair, and ten studies as good (Fig. 3). The main factors influencing the study quality was the inaccuracy of some studies in reporting data and results. The Rob 2.0 tool^14^ reported that one study was to be considered at “low risk of bias”, 53 studies with “some concerns for bias”, and 20 studies at “high risk of bias” (Fig. 4). Based on the GRADE tool, the quality of evidence of all the four primary outcomes (primary union, complications, reintervention, and failures) was judged ranging from “low” to “very low”.

## Discussion

The main finding of this meta-analysis is that different treatments offer suitable results to address the complex diaphyseal bone defects, but each one showing specific indications, strengths, and critical aspects. Despite an overall high final union rate, relatively high complication and reintervention rates were retrieved, with important differences among the various treatments, which should be considered when choosing the proper surgical approach.

The correct approach for a LDD should be chosen considering each patient individually, weighing the pros and cons of each technique, aiming to achieve safe and reproducible outcomes with low reintervention rates. The results from this meta-analysis contribute to shed some light in this direction. In particular, the first finding brought to the attention by this systematic review is the inclusion of 74 studies for a total of 1781 patients; such large numbers underline the importance of diaphyseal bone defect treatment. A lot of research efforts have been put into this field, which also shows an evolution over time. A temporal trend emerged when investigating the various treatments for LDD (Fig. 2): during the 80 s and until the mid-90 s there were only a few studies, mainly focused earlier on bone allograft or autograft and, later, on vascularized or non-vascularized fibular grafts^17,41,66,67^. Then, from the end of the 90 s, more and more studies were progressively published, along with a growing interest in other approaches such as bone transport and endoprosthesis^54,57,60–62,82–84^. Finally, in the last years, all treatments were increasingly addressed but with a focus above all on bone transport and, also due to the impulse of new augmentation procedures, with several studies on bone allograft or autografts^27–29,31,35,36,45,50,51,53,56,59,77,91^.

Traditionally, the surgical management of LDD involved bone grafting^92^, relying on the combination of mechanical stability and an osteoinductive substrate. In this meta-analysis, bone allografts or autografts have been used alone or within more advanced biological augmentation techniques. Among the retrieved studies, the mean primary union rate was 75%, an important finding both in terms of an overall good outcome, but also for the low heterogeneity of the findings, underlying the reliability and consistency of the results obtained by this approach^17–43^. Moreover, while a similar time to union and reintervention rates were found for other techniques, this approach presented one of the lowest complication and failure rates (Table 3). Interestingly, results obtained with bone allografts or autografts used alone^17–20,22,24,25,30,33^ seemed to align with those of more complex combined procedures, apart from a major rate of complications, underlining the need for better-targeted studies to demonstrate the real potential of biological augmentation techniques for cancellous or cortical-cancellous bone grafting. Finally, in the early 2000s, the Masquelet technique started to raise interest, a two-stage reconstruction technique based on cancellous bone graft and induced membrane technique^93^. Since then, the induced membrane technique was increasingly used; five studies where it was reported as a single technique could be included in this meta-analysis^28,29,36,39,40^, showing overall results comparable to those of the other autograft or allograft techniques.

The use of autologous fibular graft has been also well documented over time, either vascularized or non-vascularized. The vascularized fibula was introduced in 1975 by Taylor et al.^7^ to maximize the healing potential and bone viability while taking advantage of the possibility of treating simultaneously soft tissue damage through combined tissue flaps. In this meta-analysis only four studies used exclusively non vascularized fibula, while in the majority vascularized fibula was used, either pedicled or free. Overall, the non-vascularized fibula showed less satisfactory results in terms of primary union, complications, and reintervention rate as compared to the vascularized fibula. The use of a fibular graft proved to be a demanding surgery^94^, with the meta-analysis underlying similar results in terms of time to union and need for reinterventions, but the lower primary union and higher failure and complication rates with respect to other approaches. Moreover, the majority of complications were severe and mainly represented by fractures, which often healed at the last follow up but caused long-term discomfort and healing time. In light of these limitations, Capanna et al.^95^ combined allograft and intramedullary VFG to strengthen the construct. However, even with technique modifications and the latest developments, fibular grafts remain a challenging surgical approach^96,97^.

The bone transport technique with distraction osteogenesis with external fixation is another well-known treatment introduced in 1969 by Ilizarov for bone lesions^98^. Nowadays, it remains one of the most useful and versatile approaches to address critical-size defects^99^, with the overall higher primary union rate among the possible strategies. However, there are well-known downsides: it takes several months and a fully compliant patient to be completed, and there is a high risk of complications^100^. These aspects emerged clearly from this meta-analysis; the mean primary union rate was 91% but with a complication rate of 62%^35,36,43–65^. The large majority of complications were due to infection, especially superficial pin tract infections that often resolved without any further operative intervention^49,54,56,57,59^. Other relevant problems were ROM limitations, limb length discrepancy and deformity. To overcome these problems, in the last decades new bone transport techniques using intramedullary devices were introduced, minimizing external fixation time and joint contractures, thus increasing patient compliance and satisfaction, while achieving bone union faster^101–103^. Finally, a comparison between bifocal or trifocal bone transport was reported in one study^50^, finding the first to be quicker, more effective, and with a lower complication rate. Further RCTs are needed to confirm the potential and limitations of new bone transport techniques for the treatment of diaphyseal defects.

In the case of short life expectancy or other contraindications to biological reconstruction, intercalary endoprosthesis has been proposed for diaphyseal defects^104^. Endoprosthesis provides a valid solution when aiming for early weight-bearing, immediate stability, and fast recovery, but this comes at the price of high risks of mechanical complications such as aseptic loosening and periprosthetic fractures^33^. Therefore, the main indication is for elderly cancer patients whose healing capacity is poor and the rapid restoration of function is more important than the long term durability^11^. This was confirmed by the meta-analysis, the mean age of endoprosthesis patients was 52.7 years old, greater than that of the other techniques, and all of the patients had a diaphyseal tumour^82–86,88–90^. The complication rate was 26%, the reintervention rate 20%, and, most important, a 22% failure rate was retrieved, confirming the limits of endoprosthesis as a long-lasting solution^105^. Future improvements aiming to better prosthesis stability, such as changes in prosthesis design or better fixation methods, are needed to provide a better solution in terms of outcome and complications for these complex and fragile patients.

Different techniques are applied for the treatment of different types of patients and lesions, which could influence the results in terms of outcomes and complications. Among the four groups of techniques retrieved, the bone defect causes that led to the surgical intervention were different: bone allograft and autograft and bone transport were used mainly for traumatized patients (53.7% and 55.6%), almost all patients with a diaphyseal bone defect caused by infection were treated with bone transport techniques, while tumor resection was the indication of 73.1% of the fibular graft and 100% of the endoprosthesis (Table 2).

In this light, a specific investigation was performed to define the results of the treatment options when addressing the same etiological target. The main aetiology groups are represented by tumor and trauma (Tables 4 and 5). Concerning tumors, it is interesting to underline the lower indications for some techniques: bone transport was not applied and when bone allograft and autograft, as well as fibular grafts, were used, they offered lower results than for other treatment indications. Furthermore, this sub-analysis revealed that, despite the previously mentioned limitations, endoprosthesis still presents the lower complication and reintervention rates with respect to the other options (Table 5). Finally, a specific analysis was focused on the trauma that was the most common cause of LDD. In these patients, similar results were achieved in terms of primary union rate, but marked differences were found for different treatment options (Table 4). Fibular grafts provided a faster time to union with respect to other treatment indications and showed to gain a two-to-three months advantage versus other solutions for patients affected by trauma (Table 4). On the other hand, high complication, reintervention, and failure rates were documented in these patients, which suffered fewer complications, reinterventions, and failures when treated with bone allografts and autografts (Table 4).

This systematic review and meta-analysis included numerous studies and a high number of patients. Nonetheless, there are limitations to point out. Among all, the risk of bias and the low level of evidence of the studies, as documented by the Downs and Black’s Checklist for Measuring Quality, the Rob 2.0 tool and the GRADE system. Another limitation is the lack of prospective PROSPERO registration. Furthermore, the high heterogeneity of the included studies for defect aetiology, patient populations, and techniques, add to the literature weaknesses. In light of all this, there is a clear need for standardized, properly designed comparative studies to search for the best clinical practice in each specific scenario. However, important indications could be drawn from the meta-analysis of the available literature. First of all, this meta-analysis documented that many options are available to treat LDD, but no one appears as an optimal solution in terms of safe, satisfactory, and long-lasting outcomes, which urges the development of better surgical strategies. Moreover, many aspects have to be taken into account when choosing the most suitable approach for LDD, as results in terms of primary union rate and time to union time, as well as risks in terms of complication, reintervention, and failure rates. The results of this meta-analysis underlined the potential and limitations of the different treatment options according to the different clinical scenarios, which could help the clinicians in understanding the advantages, disadvantages, and overall, the most suitable option when treating the challenging LDD.
